# Integrated analysis of DNA methylation and gene expression profiles identified S100A9 as a potential biomarker in ulcerative colitis

**DOI:** 10.1042/BSR20202384

**Published:** 2020-12-02

**Authors:** Shasha Su, Wenjie Kong, Jing Zhang, Xinguo Wang, Hongmei Guo

**Affiliations:** 1 Department of Digestive, Traditional Chinese Medicine Hospital Affiliated to Xinjiang Medical University, Urumqi, Xinjiang 830099, P.R. China; 2 Department of Digestive, People’s Hospital of Xinjiang Uygur Autonomous Region, Urumqi, Xinjiang 830001, P.R.China

**Keywords:** integrated analysis, qRT-PCR, S100A9, ulcerative colitis

## Abstract

Ulcerative colitis (UC) is a prevalent relapsing-remitting inflammatory bowel disease whose pathogenetic mechanisms remain elusive. In the present study, colonic biopsies samples from three UC patients treated in the Traditional Chinese Medicine Hospital and three healthy controls were obtained. The genome-wide mRNA and lncRNA expression of the samples were profiled through Agilent gene expression microarray. Moreover, the genome-wide DNA methylation dataset of normal and UC colon tissues was also downloaded from GEO for a collaborative analysis. Differential expression of lncRNA (DELs) and mRNAs (DEMs) in UC samples compared with healthy samples were identified by using limma Bioconductor package. Differentially methylated promoters (DMPs) in UC samples compared with controls were obtained through comparing the average methylation level of CpGs located at promoters by using *t*-test. Functional enrichment analysis was performed by the DAVID. STRING database was applied to the construction of gene functional interaction network. As a result, 2090 DEMs and 1242 DELs were screened out in UC samples that were closely associated with processes related to complement and coagulation cascades, osteoclast differentiation vaccinia, and hemorrhagic diseases. A total of 90 DEMs and 72 DELs were retained for the construction of functional network for the promoters of their corresponding genes were identified as DMPs. S100A9, HECW2, SOD3 and HIX0114733 showed high interaction degrees in the functional network, and expression of S100A9 was confirmed to be significantly elevated in colon tissues of UC patients compared with that of controls by qRT-PCR that was consistent with gene microarray analysis. These indicate that S100A9 could potentially be used as predictive biomarkers in UC.

## Introduction

Ulcerative colitis (UC), as a relapsing chronic inflammatory disorder, is the most common form of the inflammatory bowel disease (IBD) [1]. The gastrointestinal system is a complex web of interactions among commensal bacteria, epithelial cells, resident immune cells, stromal components, recruited bone-marrow derived cells, and environmental factors. In UC patients, the delicate balance of homeostasis is disturbed for many disparate reasons [2]. UC affects not only young population, but also elderly patients [3]. It is characterized by involving only the large bowel and causing a superficial inflammation that is limited to the innermost layers with the presence of cryptitis and crypt abscesses [4]. UC tends to become a major health problem in industrialized locations including Western Europe and North America, and its incidence and prevalence continue to augment in Asia, but little is known about its exact pathogenesis [1].

Currently, it is widely accepted that UC may result from an abnormal inflammatory response to the luminal microbiota and foreign antigens in genetically predisposed subjects [5,6]. This complicated interaction of environmental, immune and genetic factors causing UC is also reflected in wide-spectrum changes in gene expression that can distinguish UC from controls [1]. These changes result in alterations in the luminal microbiota and dysregulation of the intestinal mucosal immune system, leading to the development of UC [7]. Currently, therapies targeting TNF have been relatively successful in treating certain symptoms of UC [8]. However, even with medical therapy, up to 40% of patients do not initially respond to anti-TNF therapy and approximately 30% of patients lose response over time [9]. Additionally, no less than 15% of patients will require surgery to treat UC or the disease-associated complication of dysplasia [10]. Given the high refractory response rates, and the expanding clinical need, diagnostic marker and new therapy targets are still needed to treat UC.

To date, a lot of the previous work on UC pathophysiology has focused on the associated protein-coding transcripts. For example, the leucine-rich alpha-2 glycoprotein level in serum was identified as a potential UC biomarker, which was associated with the disease activity [11]. LncRNAs are also found to link to a plethora of human pathologies, including inflammatory diseases [12]. So far as UC is concerned, several lncRNAs have been recently identified to be differentially expressed among active UC, UC in remission and healthy controls. Among the multiple dysregulated lncRNAs, IFNG-AS1 was found to be associated with single-nucleotide polymorphism (SNP) rs7134599 and locates near the inflammatory cytokine interferon-γ (IFN-γ). INFG-AS1 was found to positively regulate IFNG expression, confirming the crucial role of lncRNAs in inflammatory cascades related to UC [4,13]. Additionally, compared with the controls, elevated level of lncRNA H19 was observed in UC patients [14]. Similarly, another lncRNA, BC012900, was found to be significantly differentially expressed in active UC tissues compared with other conditions and stimulated by pathogens and cytokines through known UC signaling pathways like NOD2 receptor and Toll-like [15]. However, to our knowledge, few researches have taken both of mRNA and lncRNA expression and DNA methylation into account when screening for UC-related biomarkers.

**Figure 1:**
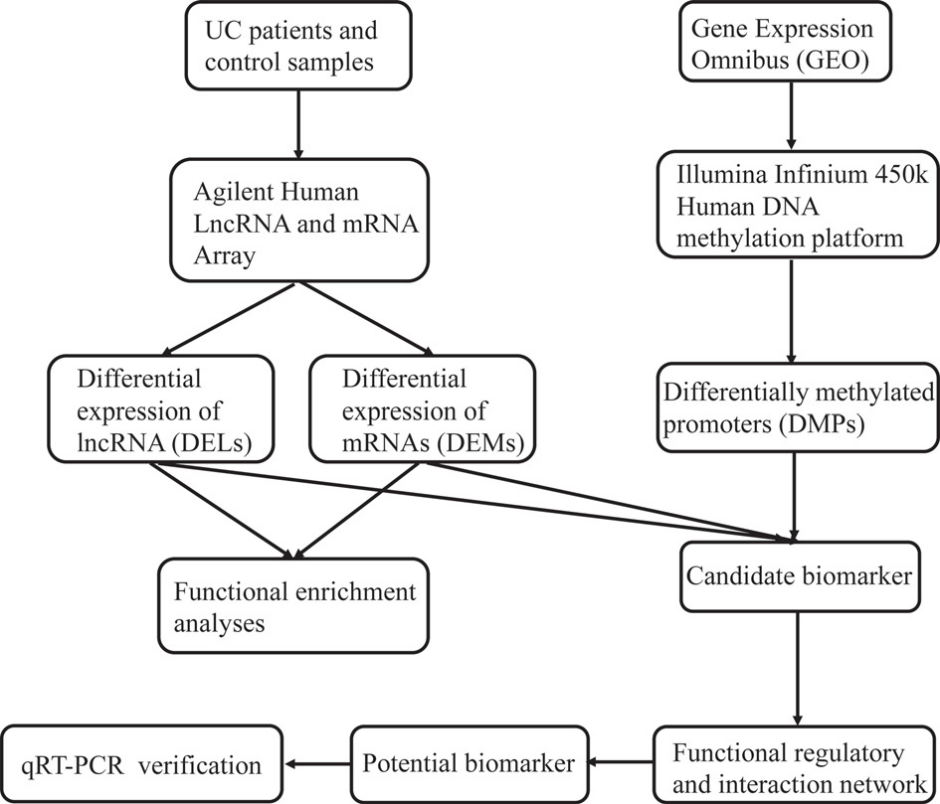
The flow chart of the present study

The flow chart of this research is shown as Figure 1. The aim of the present study is to screen novel mRNAs and lncRNAs signatures for the diagnosis and therapy of UC. To explore the transcriptomic profiles of UC patients, 3 controls and 3 active UC samples were obtained from the patients treated in the Traditional Chinese Medicine Hospital and analyzed using gene expression microarrays. Through integrated analysis of the differentially methylated CpGs in the promoter region of corresponding genes, multiple candidate differentially expressed mRNAs and lncRNAs were screened and an integrated regulatory network was constructed. The mRNAs and lncRNAs with high degree in the network were selected and their differential expressions between UC and control samples were further validated by quantitative real-time reverse-transcription polymerase chain reaction (qRT-PCR). The present study should be helpful to provide potential biomarkers for UC.

## Materials and methods

### Clinical samples

A total of three UC patients that diagnosed on the basis of clinical symptoms and histological examination consistent with the disease in the Traditional Chinese Medicine Hospital were included in the present study. Samples from normal controls and UC patients with active endoscopic inflammation were obtained by colonic biopsy from the left colon. Written informed consent of the patients was provided by their legal surrogates to permit surgical procedures and use of resected tissues. The present study was approved by the Specialty Committee on Ethics of People’s Hospital of Xinjiang Uygur Autonomous Region.

### RNA extraction

Total RNA was extracted from the biopsies of UC patients and controls using TRIzol (Qiagen, Valencia, CA) and miRNeasy Mini Kit (Qiagen), according to the manufacturer’s instructions. The purity and quantity of extracted RNA were determined by UV absorbance using a NanoDrop 1000 Spectrophotometer (Thermo Scientific), and the integrity of RNA was assessed with the Agilent Bioanalyzer RNA 6000 LabChip kit (Agilent Technologies).

### Gene expression microarray

The lncRNA and mRNA expression profiles were determined by Agilent Human LncRNA + mRNA Array v4 (4 × 180K). For the microarray, three controls and three active UC samples were used, involving 72,771 RNAs (38,141 lncRNAs, 31,705 mRNAs, and 2925 other RNAs).

### Methylation data source

The methylation dataset of UC (GSE81211) consisting of three normal colon samples from healthy controls and eight colon samples from active UC patients was downloaded from the Gene Expression Omnibus (GEO) of the National Center for Biotechnology Information [16]. The DNA methylation profiling was assessed by Illumina Infinium 450k Human DNA methylation platform, including more than 480,000 CpGs.

### Identification of differentially expressed genes

Differential expression analysis was conducted between the disease group and the control group. Limma Bioconductor package was used for differential analysis of quartile normalized expression profiles. Differential expression of lncRNA (DELs) and mRNAs (DEMs) were defined according to the following criteria: |log2(Fold Change)| >1 and adjusted *P*-value (FDR) <0.05.

### Definition and classification of promoter regions

The promoter region was defined as 1500 bp upstream to 500 bp downstream of the transcription start site (TSS). Promoters were classified into three groups consisting of high-CpG promoters (HCP), intermediate CpG promoters (ICP), and low-CpG promoters (LCP) based on the CpG ratio, CpG content, and CpG region length. HCPs were defined as promoters containing at least one 500 bp-region with CpG ratio >0.75 and GC content >55%, while LCPs were defined as promoters without a 500 bp-region with CpG ratio >0.48. ICPs were those not classified into either HCP or LCP. For the corresponding genes of mRNAs, 1786 HCP, 5939 ICP and 19,637 LCP were included. While for the corresponding genes of lncRNAs, 6128 HCP, 5361 ICP and 22,602 LCP were included.

### Identification of differentially methylated promoter

The methylation level of a gene promoter region is calculated by averaging the methylation level of all CpG sites in its promoter region. Differentially methylated promoters (DMPs) were defined as the promoters with differential methylation that ranked the highest and lowest 5% between UC patients and healthy controls in *t*-test.

### Functional enrichment analyses

Functional enrichment analysis of differentially expressed mRNAs was conducted by Database for Annotation, Visualization and Integrated Discovery (DAVID) (https://david.ncifcrf.gov/) [17]. For functional enrichment analysis of differentially expressed lncRNAs, GREAT was employed to annotate the genes regulated by the lncRNAs [18]. Adjusted *P*-value < 0.05 was used as the threshold for significant enrichment.

### Construction of functional regulatory and interaction network

In order to establish the functional regulatory network between lncRNAs and mRNAs, the correlation of lncRNA and mRNA expression was calculated. Only the lncRNA–mRNA pairs with expression correlation above 0.98 and *P*<0.01 were defined as true regulatory pairs and used for constructing functional regulatory network. Protein–protein interaction information in STRING database was employed to extract the functional module of candidate marker from the functional regulatory network [19].

### qRT-PCR

The expressions of several differentially expressed genes from microarray experiments were validated by qRT-PCR, with GAPDH as the internal reference. All cDNAs were prepared using 500 ng of RNA using TaqMan reverse transcription reagents with a mixture of random and oligo(dT) primers. Realtime PCR was performed in triplicate using the SYBR Green mastermix (Bio-Rad) in ABI PRISM 7500 sequence detection system (Applied Biosystems). For PCR amplification, the following thermal profile was used by an additional denaturation step at 95°C for 15 s, annealing at 60°C for 1 min, and with a slow increase in temperature back to 95°C with a ramp time of 19 min 59 s to ensure amplification of the correct genes. The relative expressions were calculated using 2-ΔΔCt method. The primer sequences were shown in Table 2.

### Construction of the logistic regression model

The datasets of GSE87473 (consisting of 106 colonic epithelial mucosal biopsy samples of UC patients and 21 control samples of healthy people) and GSE48634 (consisting of 68 colonic epithelial mucosal biopsy samples of UC patients and 69 control samples of healthy people) were downloaded from the Gene Expression Omnibus (GEO) of the National Center for Biotechnology Information [16]. The datasets were assessed by the platforms of Affymetrix HT HG-U133+ PM Array Plate and the Illumina HumanHT-12 V4.0 expression bead chip, respectively.

To screen reliable UC biomarkers, a logistic regression model was constructed using sample type, i.e. UC or normal, as response variables and the mRNA expression levels as predict variables in the training set (GSE48634) by using the glm function in R language. Furthermore, the sample types in GSE87473 as testing sets were predicted through the CRC diagnostic model. Receiver operating characteristic curve (ROC) was plotted and area under ROC (AUC) was calculated for evaluating the performance of the model.

## Results

### RNA type landscape in the microarray

**Figure 2:**
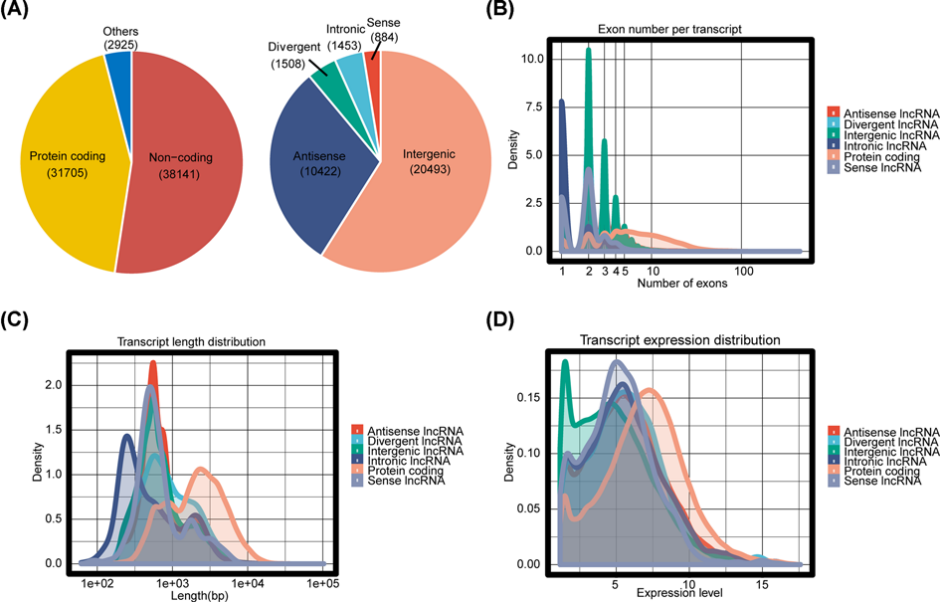
Analysis of the types and properties of RNAs with CapitalBio-chip array (**A**) The percentage of different types of RNAs. (**B**) Distribution of exon number per transcript. (**C**) Transcript length distribution. (**D**) Transcript expression distribution.

Expressions of a total of 72,771 RNAs, including 38,141 lncRNAs, 31,705 mRNAs and 2925 other RNAs were profiled in the microarray as shown in the left panel of Figure 2A. Right panel of Figure 2A illustrated the detailed information of numbers of different lncRNA types among the 38,141 lncRNAs. Exon number (Figure 2B), transcript length (Figure 2C) and expression level (Figure 2D) of mRNAs were much higher than those of lncRNAs. Specifically in lncRNAs, the exon number per transcript in lincRNAs and antisense lncRNAs was higher than that in other lncRNAs (Figure 2B), while transcript length of intronic lncRNAs was much shorter and the expression level of lincRNAs was generally lower than that of other lncRNAs (Figure 2C,D).

### DEMs and DELs

**Figure 3:**
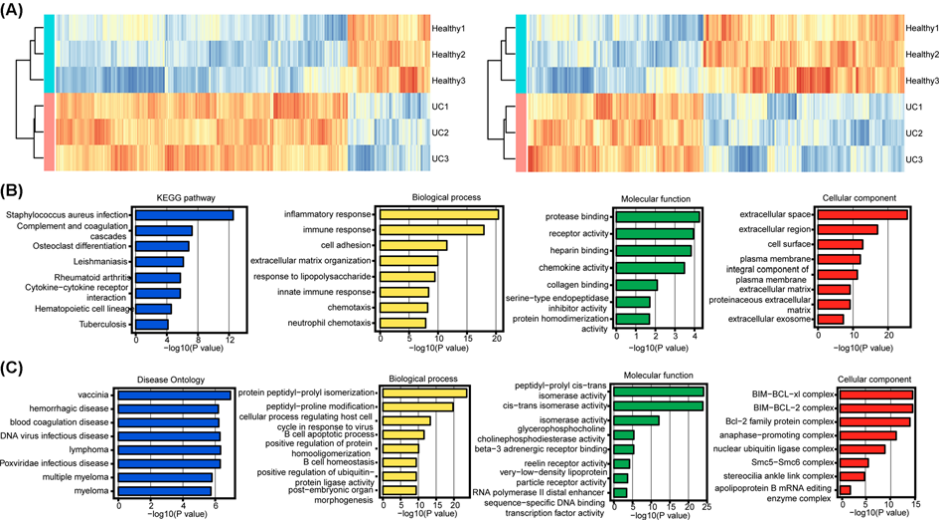
Functional enrichment analysis of differentially expressed mRNAs and lncRNAs (**A**) Clustering of differentially expressed mRNAs and lncRNAs. (**B**) Functional enrichment analysis of differentially expressed mRNAs. (**C**) Functional enrichment analysis of differentially expressed lncRNAs.

A total of 2090 DEMs and 1242 DELs were identified in UC samples compared with healthy controls. Figure 3A left and right panel shows the hierarchical clustering results based on the expressions of DEMs and DELs, respectively. UC and healthy samples could be accurately distinguished from each other.

### Functional enrichment analysis

Functional enrichment analysis was performed on DEMs, and they were significantly enriched in KEGG pathways, including complement and coagulation cascades and osteoclast differentiation pathways. The significantly enriched GO terms were converged on biological processes of cell adhesion and extracellular matrix organization, molecular functions of protease binding and receptor activity, and cellular components of extracellular space and extracellular region (Figure 3B). The differentially expressed lncRNAs were significantly enriched in vaccinia and hemorrhagic diseases, biological processes of protein peptidyl-prolyl isomerization and peptidyl-proline modification, molecular functions of peptidyl-prolyl cis–trans isomerase activity and cis–trans isomerase activity, and cellular components of BIM–BCL–xl complex and BIM–BCL-2 complex (Figure 3C).

### Methylation landscape of different promoter types

**Figure 4:**
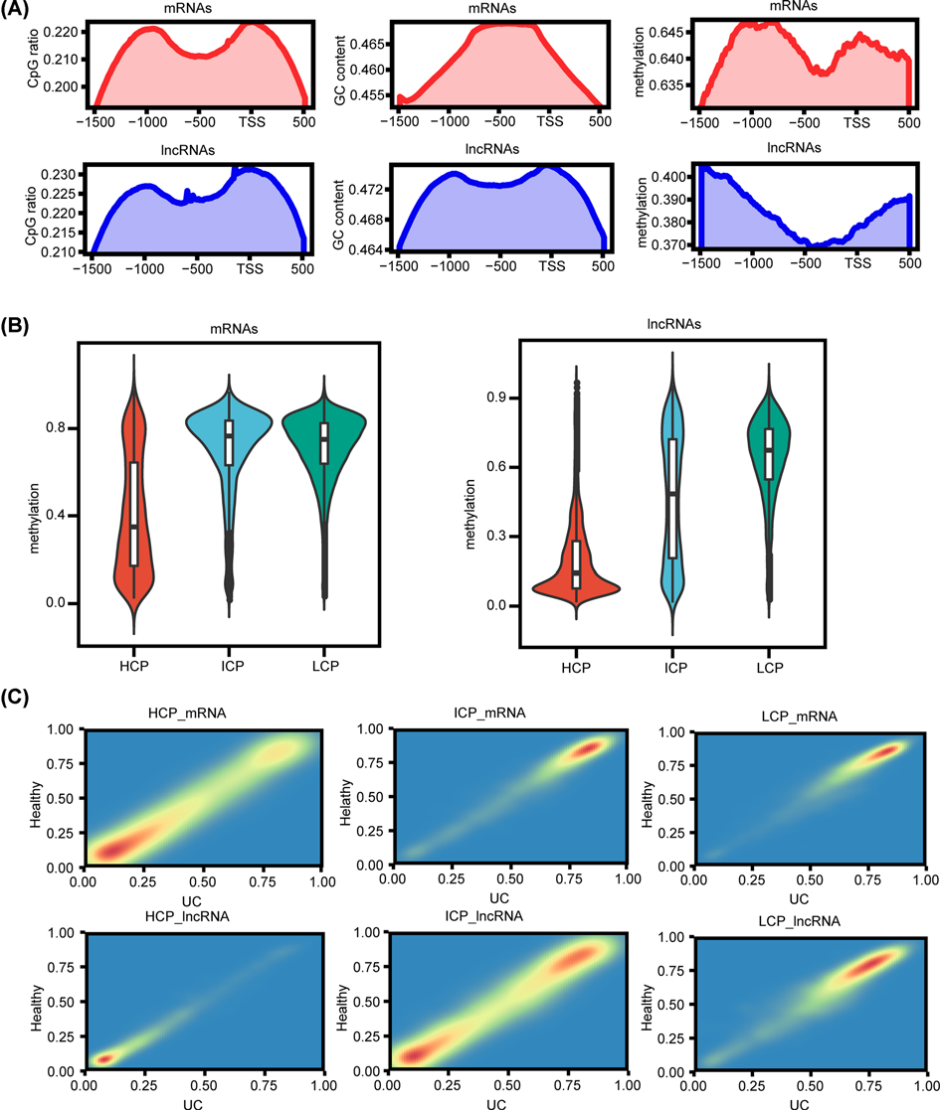
Methylation analysis of mRNA and lncRNA promoters with different CpG densities (**A**) CpG ratio, GC content and methylation level distribution in mRNA and lncRNA promoter regions. (**B**) Comparison of HCP, ICP and LCP methylation levels of mRNA and lncRNA. (**C**) Comparison of methylation level of HCP, ICP, LCP in mRNA and lncRNA between UC and control groups.

Promoter region of both mRNA and lncRNA corresponding genes had the highest CpG density (including CpG ratio and GC content) near TSS, and there was a negative correlation between methylation level and CpG density (Figure 4A). These promoters were divided into three categories: HCP (high-CpG promoters), LCP (low-CpG promoters) and ICP (intermediate-CpG promoters) based on the density of CpG. The methylation levels of these promoters were different. HCP had the lowest methylation level, while LCP had the highest methylation level, which was especially obvious in corresponding genes of lncRNAs (Figure 4B). By comparing the methylation levels of mRNA and lncRNA corresponding genes’ promoters in the disease and control groups, it was found that the methylation levels HCPs of mRNA and lncRNA corresponding genes were relatively low in both disease and control groups, LCPs of mRNA and lncRNA corresponding genes and ICPs of mRNA corresponding genes showed high methylation levels in both disease and control samples, while ICPs of lncRNA corresponding genes showed obvious polarization of the methylation level (Figure 4C).

### Integrated analysis of gene expression and DNA methylation datasets

**Figure 5:**
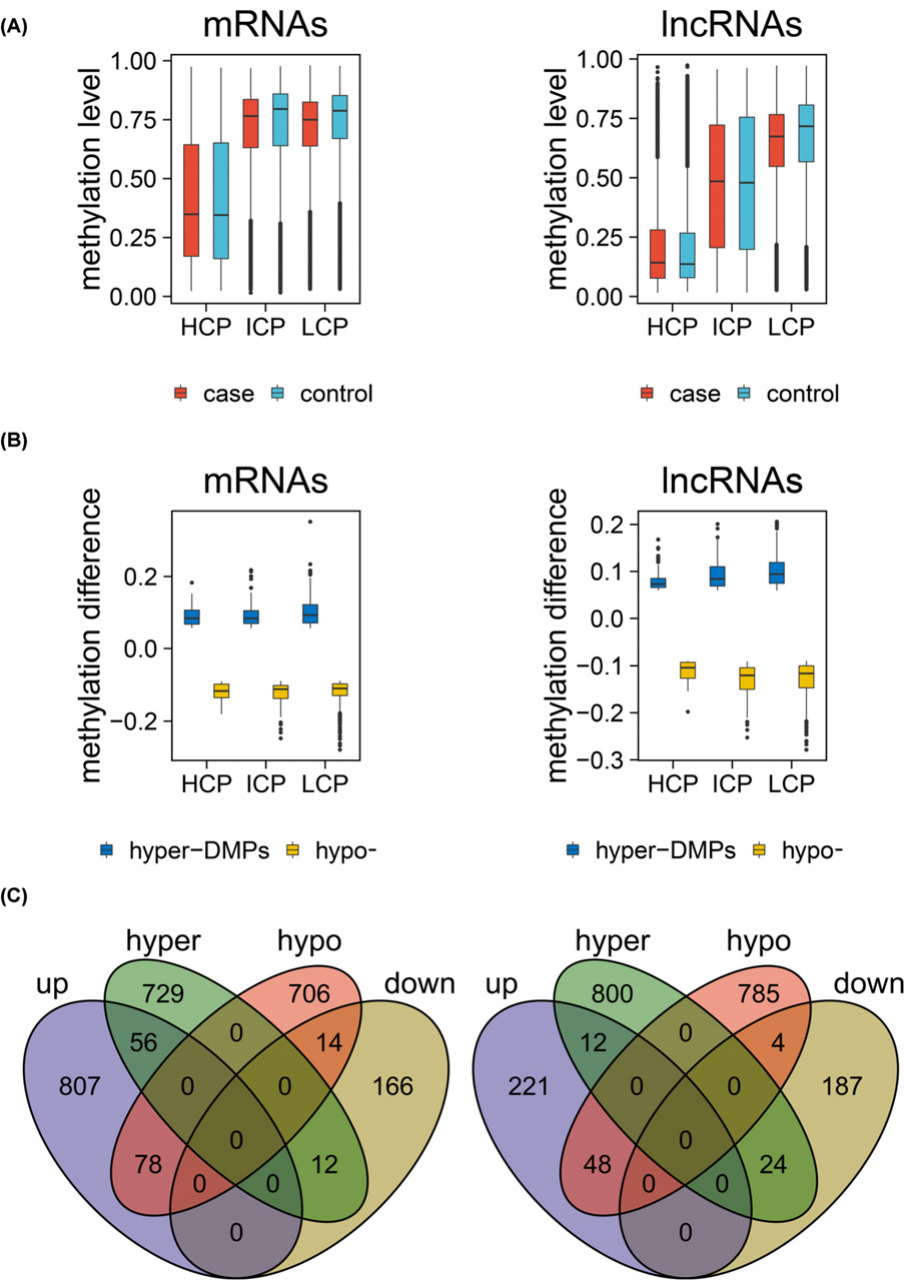
Analysis of differentially methylated mRNA and lncRNA promoters (**A**) Comparison of methylation levels of different promoters between UC and control groups. (**B**) Differences in methylation of different promoters between UC and control groups. (**C**) Integrated comparison of differential expression and differential methylation.

A total of 1595 promoters of mRNA corresponding genes and 1673 promoters of lncRNA corresponding genes were identified as DMPs in UC samples compared with healthy samples (Table 1). In both mRNA and lncRNA corresponding genes, methylation levels of ICP and LCP were higher than that of HCP (Figure 5A), and the difference of methylation of LCP between UC and control group was higher than that of HCP and ICP (Figure 5B). There were 12 down-regulated mRNAs with hyper-methylated promoters and 78 up-regulated mRNAs with hypo-methylated promoters, while 24 down-regulated lncRNAs with hyper-methylated promoters and 48 up-regulated lncRNAs with hypo-methylated promoters in the corresponding genes (Figure 5C). Those RNAs were considered as candidate markers for the construction of subsequent functional networks.

**Table 1:** Statistics on cross-comparisons between promoter subcategories and methylation subcategories

class	hil	total	hyper-DMPs	hypo-DMPs
lncRNA	HCP	6067	119(1.96%)	56(0.92%)
lncRNA	ICP	3994	256(6.41%)	142(3.56%)
lncRNA	LCP	6669	461(6.91%)	639(9.58%)
mRNA	HCP	1756	76(4.33%)	21(1.2%)
mRNA	ICP	4413	192(4.35%)	135(3.06%)
mRNA	LCP	9779	529(5.41%)	642(6.57%)

**Table 2:** Primers for qRT-PCR

Gene	Sequences	Length (bp)
S100A9 Forwad	5′- CATGGAGGACCTGGACACAAA -3′	103
S100A9 Reverse	5′- CTCGTGCATCTTCTCGTGGG -3′	
HECW2 Forwad	5′- AGAACTGATTGCTCTCCTGTGA -3′	82
HECW2 Reverse	5′- GCAGGAGTGTAACATAAGTGGTA -3′	
HIX0114733 Forwad	5′- TGTTGAGGCGACTGATAAGGG -3′	113
HIX0114733 Reverse	5′- ACAGAGAGGTCTGGTTGGGG -3′	
SOD3 Forwad	5′- ATGCTGGCGCTACTGTGTTC -3′	99
SOD3 Reverse	5′- CTCCGCCGAGTCAGAGTTG -3′	
GAPDH Forwad	5′- AACGACCACTTTGTCAAGC -3′	73
GAPDH Reverse	5′- TGAGGTCCACCACCCTGT -3′	

Forward: Forward primer; Reverse: Reverse primer

### Functional regulatory and interaction network

**Figure 6:**
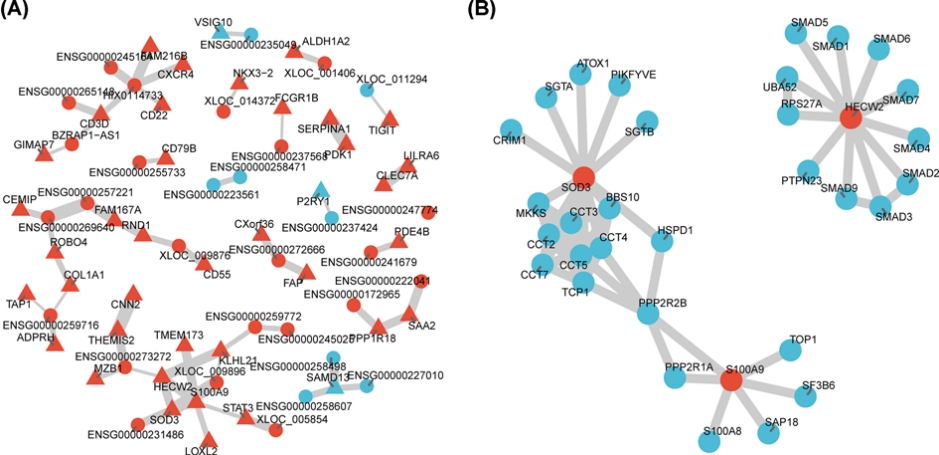
Construction of integrated regulatory network (**A**) A functional network of candidate markers based on co-expression and methylation levels. (**B**) Function modules of candidate markers based on protein interaction network.

Based on the integrated analysis of expression and methylation, 162 candidate markers (90 mRNAs and 72 lncRNAs) were finally obtained, and their functional regulatory network was constructed through co-expression analysis. Figure 6A illustrated the lncRNA–mRNA regulatory network. It was shown that three mRNAs, S100A9, HECW2 and SOD3 had high degrees in the network, and whose functional interaction modules centering on the three mRNAs was obtained based on the STRING database (Figure 6B).

### Validation of the differential expression of potential biomarkers

**Figure 7:**
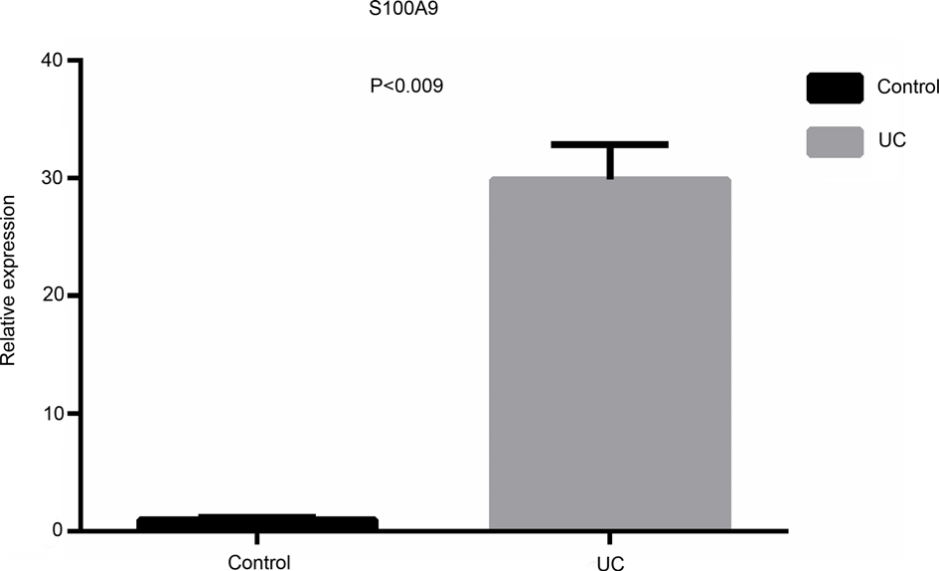
Real-time reverse transcription-PCR (qRT-PCR) verification of S100A9 expression

We analyzed the expressions of S100A9, HECW2 and HIX0114733 in a separate cohort by qRT-PCR in colonic biopsies obtained from healthy controls and UC patients. The samples consisted of five UC samples and five control colonic biopsy samples. The results showed that HECW2 and HIX0114733 expression levels did not attain a statistically significant difference between UC and control samples (Supplementary Figure S1), while an obvious increase of S100A9 expression level in UC-active patients was observed compared to controls (*P*=0.009) (Figure 7), which was consistent with the result of gene expression microarray.

### Construction and evaluation of the logistic regression model

The expressions of S100A9, HECW2 and HIX0114733 were used as predict variables in the logistic regression model. The constructed model conformed to a normal distribution (Supplementary Figure S2A), and the predict variables included in the model had a good linear relationship with the response variable (Supplementary Figure S2B). There are no extreme points that significantly affect the accuracy of the model (Supplementary Figure S2C). Moreover, AUC of the model could achieve 0.67 and 0.88 when applied it to the training set and the testing set respectively (Supplementary Figure S2D), which illustrated the good performance of the model in UC diagnostic.

## Discussion

The routine diagnosis for UC includes clinical symptom assessment combined with endoscopic examination, serology, radiology, and histology. Serologic markers have also been evaluated as a means to diagnose UC noninvasively. However, there is no single gold standard test available for diagnosing UC [15,20]. Currently, RNAs are better understood in sorts of cancers and auto-immune diseases, such as UC. Hundreds of mRNA molecules are shown to be deregulated in UC patients and some of them indicate molecular disease mechanisms that remain to be confirmed. Aside of them, LncRNAs, accounting for roughly 10% of human genome [21], have been implicated in important biological processes such as carcinogenesis [4], pluripotency [15], cell-cycle progression [4], apoptosis [22] and cellular senescence. For antisense lncRNAs, they are associated with substrate pools of different RNA degradation pathways and enzymes. Additionally, chromatin-associated RNAs (CARs) – both intronic and intergenic – form an integral component of chromatin, with the potential to regulate the expression of nearby genes.

To explore the transcriptome profiles of UC patients and screen novel mRNAs and lncRNAs signatures for the diagnosis and therapy of UC, 3 controls and 3 active UC samples were analyzed using gene expression microarrays. Through an RNA expression analysis, we identified 2090 mRNAs and 1242 lncRNAs that are differentially expressed between UC samples and controls. The functional annotation analysis Functional annotation analysis performed on these DEMs and DELs identified multiple GO term and KEGG pathways, such as complement and coagulation cascades, cell adhesion and extracellular matrix organization, vaccinia and hemorrhagic diseases and protein peptidyl-prolyl isomerization and peptidyl-proline modification. Previous studies have demonstrated that the aforementioned GO terms are potentially important events in the pathogenesis of UC. For instance, cell adhesion, extracellular matrix organization, protease binding and receptor activity have roles in the pathogenesis of UC [23]. Furthermore, module analysis further confirmed that common significant pathways involved in the pathogenesis of UC were associated with hemorrhagic disease, protein peptidyl-prolyl isomerization, peptidyl-proline modification, peptidyl-prolyl cis–trans isomerase activity and cis–trans isomerase activity [24,25].

DNA methylation is one of the most important epigenetic mechanisms that regulate gene expression. In addition to sequence variation, it is gradually accepted that this DNA modification may be involved in the susceptibility of various multifactorial diseases. We analyzed global differences in methylation profiles and the degree of difference in methylation level of each site in terms of location (the distance from transcription start site and promoter type). In both mRNA and lncRNA corresponding genes, methylation levels of ICP and LCP were higher than that of HCP, and the difference of methylation in LCP was higher than that in HCP and ICP. Previous studies have confirmed that that LCPs are generally associated with tissue-specific genes, whereas HCPs are associated with two classes of genes, including highly regulated ‘key developmental’ genes and ubiquitous ‘housekeeping’ genes [26,27]. By integrating DNA methylation and gene expression profiles, we observed 12 down-regulated mRNAs with hyper-methylated promoters, 78 up-regulated mRNAs with hypo-methylated promoters, 24 down-regulated lncRNAs with hyper-methylated promoters and 48 up-regulated lncRNAs with hypo-methylated promoters in their corresponding genes. A total of 90 mRNAs and 72 lncRNAs were considered as candidate markers for the construction of functional regulatory network. It was shown that three mRNAs, S100A9, HECW2, SOD3, and lncRNA HIX0114733 exhibited high interaction degrees in the network. Moreover, qRT-PCR further validated that S100A9 was highly expressed in UC samples compared with the healthy controls. Previous analyses of the immune cell activation pathways have demonstrated that S100A9 are immunologically important. For instance, it has been found that in large intestinal epithelial cells, the STAT3 pathway is stimulated by IL-6, resulting in excessive secretion of S100A9 [28], and in a study of colon cancer pathogenesis involving RAGE knockout mice, S100A9 was found to play an important role in the progression from chronic inflammation to cancer by activating the RAGE-NF-kB pathway [29]. Therefore, S100A9 might act as crucial mediators inside/outside of activated immune cells assisting such cells in self-catalytically modulating their immunologic functions through undefined autocrine pathways. Several independent evidences also indicate that S100A9 is implicated in progressions of active UC and future researches are required to underlying their potential mechanisms [30].

In conclusion, the present study used an integrated analysis method to identify DEMs and DELs, as well as biological functions and pathways in UC, thereby enhancing the current understanding of the pathogenesis and the molecular mechanisms of UC. In addition, these results may provide potential biomarkers for the differential diagnosis of UC, as well as potential therapeutic targets for the development of novel treatments. However, the present study only included a bioinformatics analysis and qRT-PCR experiment. Further experiments and analyses of larger sample size are required to confirm the ability of the above candidate genes in UC.

## Supplementary Material

Supplementary Figures S1-S2

## Data Availability

The data that support the findings of the present study are uploaded in public datasets. For more information, please see and download with the link: https://www.ncbi.nlm.nih.gov/geo/query/acc.cgi?acc=GSE160804.

## Abbreviations

DEL: differential expression of lncRNA
DEM: differential expression of mRNA
DMP: differentially methylated promoter
HCP: high-CpG promoters
IBD: inflammatory bowel disease
ICP: intermediate CpG promoters
LCP: low-CpG promoters
TSS: transcription start site
UC: ulcerative colitis
