# Supplementary figures and images for: Integrated analysis of DNA methylation and gene expression profiles identified S100A9 as a potential biomarker in ulcerative colitis
Source: Biosci Rep. 2020 Dec 2;40(12):BSR20202384. doi: 10.1042/BSR20202384 (PMC7711060; doi:10.1042/BSR20202384)

Figure S1

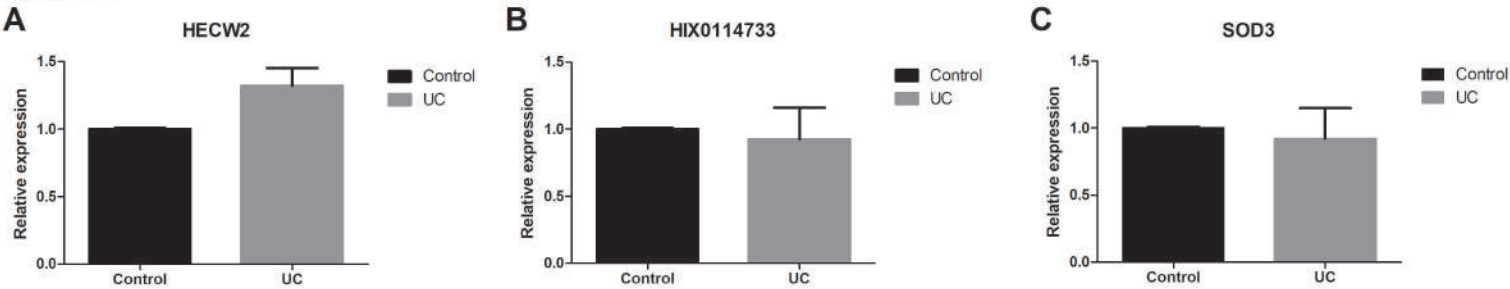

Figure S2

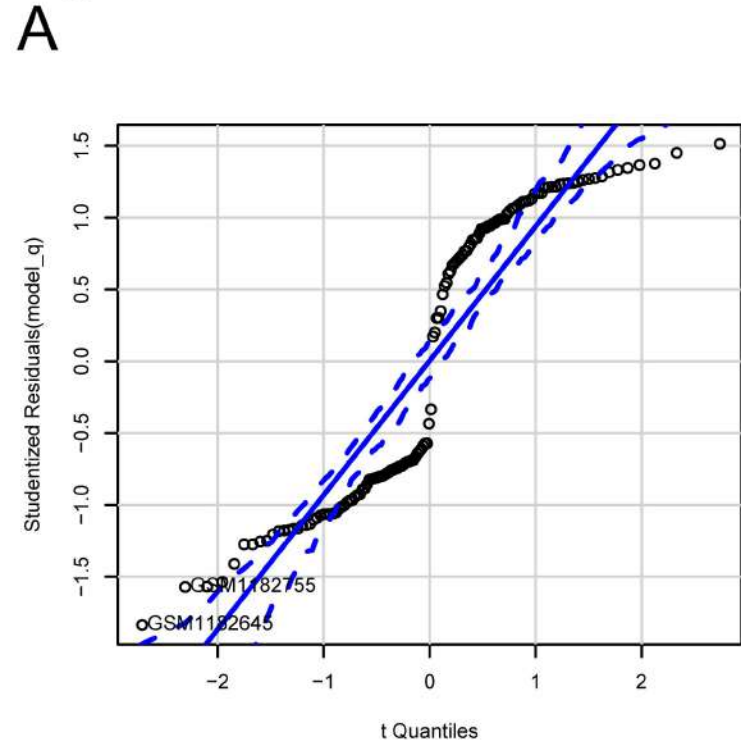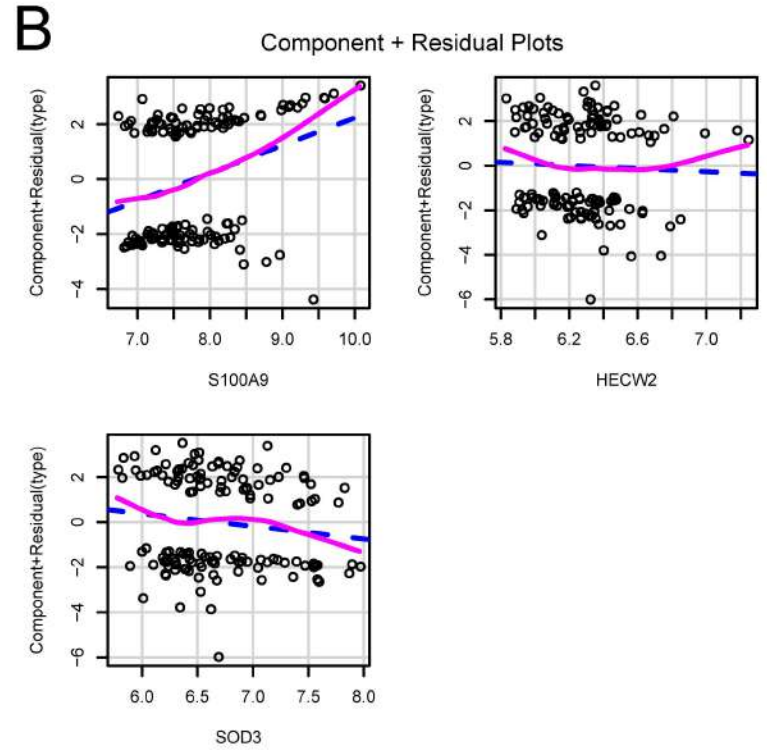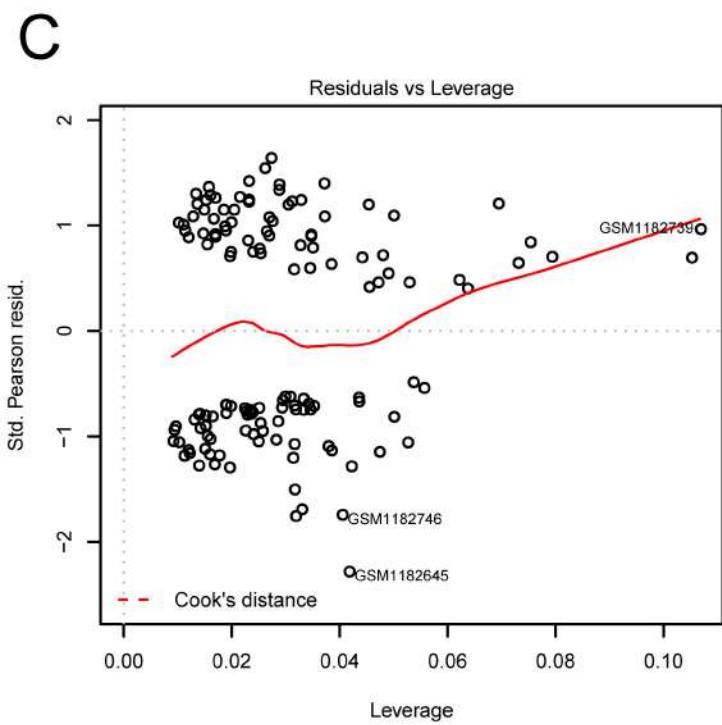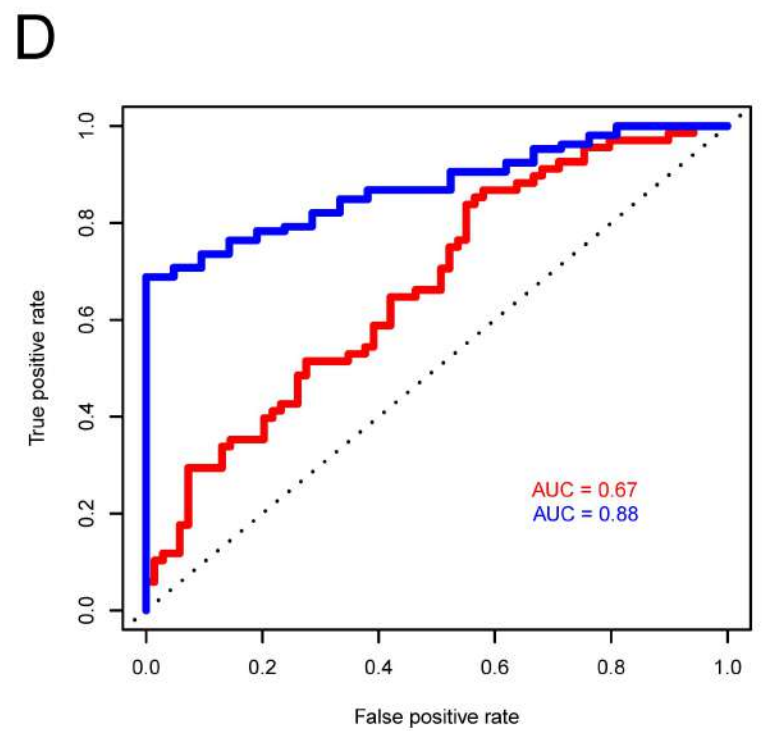

Supplement: Supplementary Figures S1-S2 [file BSR-2020-2384_supp.pdf]
